# A Multilevel Analysis of Organizational Support on the Relationship between Person-Environment Fit and Performance of University Physical Education Teachers

**DOI:** 10.3390/ijerph17062041

**Published:** 2020-03-19

**Authors:** Chia-Ming Chang, Li-Wei Liu, Huey-Hong Hsieh, Ko-Chia Chen

**Affiliations:** 1 Department of Physical Education, Health & Recreation, National Chiayi University, Chiayi 62103, Taiwan; gr5166@yahoo.com.tw; 2 Department of Leisure Service Management, Chaoyang University of Technology, Taichung 41349, Taiwan; ijofsrm@gmail.com; 3 Department of Leisure Management, Taiwan Shoufu University, Tainan 72153, Taiwan; 4 Office of Physical Education Academic Affairs, National Pingtung University of Science and Technology, Pingtung 912, Taiwan; kochia@mail.npust.edu.tw

**Keywords:** university teachers, physical education, work performance, organizational support

## Abstract

Limited research has evaluated the performance of physical education (PE) teachers. This study aimed to use person-environment fit and organizational support to evaluate PE teachers’ work performance using multilevel analysis. The relationship between person-environment fit and performance of university physical education teachers (at the person-level) and a cross-level effect on performance of university physical education teachers of perceived organizational support (at the school-level) and a moderator effect of organizational support were examined. A total of 447 PE teachers recruited from 55 universities in Taiwan were invited to participate in this survey, with a return rate of 65.74%. Using hierarchical linear modeling, the study found that person-job fit, person-organization fit, and person-supervisor fit at individual level have positive impacts on the performance of university PE teachers. As for cross-level effect, organizational support has positive impacts on the performance of university physical education teachers. However, organizational support at school level had no significant moderating effects on the relationship between person-environment fit and the performance of university physical education teachers. The implications of the findings for both university PE teachers and administrators and suggestions for future research are discussed.

## 1. Introduction

Today, the scarce resource for a company is no longer money. It is human ability. Despite the fact that businesses have placed considerable attention to work performance evaluation, it not until 1990 that performance evaluation began to receive proper attention, especially in the field of sports. Performance evaluation has been practiced mostly in professional settings, and some examples include performance evaluation of sports coaches [1], professional baseball players [2], professional golf players [3], NBA teams across the league [4], and the performance analysis of table tennis at the 2012 Summer Olympics in London [5]. Some universities in Taiwan also apply this approach to measure the performance of physical education (PE) departments [6], PE teachers [7], and university instructor’s research capability [8]. As for universities’ administration, the most important step in providing your people with development is to understanding how they are currently working and where they need to improve. However, very limited research has evaluated the performance of university faculty members, and is especially rare for PE teachers in Taiwan. Therefore, it is notable that a thorough understanding of the theory and practice of performance evaluation is required in the area of research to assist the administrators to better understand PE teachers’ performance and provide necessary support for them.

Early empirical studies on university PE teachers’ job performance have put much emphasis on performance evaluation. Very few have discussed factors that affect their job performance. Knowing the crucial factors affecting workers’ performance would provide useful information to assist workers and human resources to increase productivity and performance.

Speaking of work performance-related factors, the environment employees surrounded is an important factor which may affect their performance. Person-environment fit (P-E fit) is one among the factors related to work performance. P-E fit is defined as the degree to which individual and environmental characteristics match [9]. Based on Jansen and Kristof-Brown’s [10] theory, P-E fit contains four dimensions: person-job fit (P-J fit), person-group fit (P-G fit), person-organization fit (P-O fit), and person-supervisor fit (P-S fit). Previous studies indicated that person-environment fit is a significant resource that not only helps an organization recruits the ideal job candidates, but also improves organizational efficiency and maintains organizational competencies [11]. For instance, a meta-analysis of Hoffaman and Woehr [12] indicated that P-E fit has a predictive effect on job performance, organizational citizenship behavior (OCB), and intention to leave the current job. Their research results suggest that P-E fit is positively related to job performance and organizational citizenship behavior, but negatively related to intention to quit the job. Similarly, recent empirical work with psychological therapists and lab technicians found that a higher degree of P-E fit results in higher job satisfaction [13,14]. The study findings of Nabil [15] with academic members at the Canadian International College of Egypt have a similar conclusion that higher P-O fit can reduce emotional exhaustion at work. In summary, a review of extant literature has suggested that P-E fit can positively affect work attitudes and behaviors of skilled professionals.

Another important factor related to work performance is organizational support. According to Eisenberger, Huntington, Hutchison, and Sowa [16], perceived organizational support affects employee performance in three different ways: (a) employees have a sense of responsibility to help the organization to complete tasks, (b) employees are emotionally attached and committed to the organization, and (3) employees expect to be recognized and awarded based on their job performance. Liao, Joshi, and Chuang [17] discovered that employees are more likely to give back to the organization if they perceive high levels of organizational support and feel like being an “insider”. However, despite the fact that employee involvement does not necessarily result in high job performance due to their limited capability and resources, they are willing to cooperate with others and be actively engaged. Furthermore, Rhoades and Eisenberger [18] conducted a meta-analysis based on 70 studies on perceived organizational support and job performance, and found a positive and significant relationship between two variables.

Thus, this study first aims at exploring the relationship between university PE teachers’ job performance and four dimensions (P-J fit, P-G fit, P-O fit, and P-S fit) of P-E fit. Furthermore, this study will conduct a cross-level analysis to explore the impacts of the group level‘s (or school level’s) organizational support on university PE teachers’ job performance. Last but not least, this study also explores whether perceived organizational support has a moderating effect on the relationship between P-E fit and university PE teachers’ job performance.

## 2. Literature Review and Hypothesis Development

This section will review the literature of the abovementioned items and postulate hypotheses accordingly. First, we will start to examine relationships of the four dimensions of person-environment fit and performance. Second, we will examine previous studies related to organizational support and work performance. Third, we will examine the moderating effects of organizational support on the relationship of persona-environment fit to performance based on previous studies and then we will combine the theories and studies and present the research framework.

### 2.1. Person-Environment Fit

#### 2.1.1. Person-Job Fit

Previous studies have indicated that person-environment fit (P-E fit) is an outcome of people-environment interaction. A good fit creates good behavior [10,12,19]. A good P-E fit, for example, reduces workplace conflict [20], boosts employee motivation, and increases their commitment [21]. In this situation, employees tend to be satisfied with their job and have best chances of career success [22]. Moreover, Edwards [23] stated that person-job fit (P-J fit) refers to the degree of fit between individual skill and job requirement. Individual skills include knowledge, work skill, and experience; on the other hand, job requirements are workload and job performance.

A good match of individual skill to the job requirement is typically a decisive factor in finding a right job candidate, as this match directly affects employee and organization performance [24]. Afsar, Badir, and Khan [25] studied employees from knowledge-based industries (electronics, pharmaceuticals, and InfoTech) and their empirical evidence suggested that positive perceptions of P-J fit result in positive work behaviors. Accordingly, Hypothesis H1-1 was proposed as follows:

**Hypothesis** **H1-1.** *Person-job fit will have a positive impact on university PE Teacher performance.*

#### 2.1.2. Person-Group Fit

Person-group fit (P-G) is the fit of degree between the individual and the work team they belong to [26]. Group is defined as a small organization which includes colleagues from either a single department or cross-department [26]. Moreover, a work team is cross-functional, and therefore is within the scope of person-group fit.

For heterogeneous studies, a work team of a group of people with various skills and experience is more effective at work [27]. By contrast, from a homogeneous perspective, individuals and teams as a whole work together better if core values and goals are equally shared and appreciated within the team [28]. The social identity theory of Tajfel and Turner [29] stated that individuals define themselves according to the society they are in. In other words, individuals feel a sense of belonging to a group and feel valued and emotionally attached. However, a lack of communication and trust leads to reduced individual job satisfaction and team performance [30]. Werbel and Gilliland [31] reported that a good P-G fit increases team effectiveness and organization performance. Thus, Hypothesis 1−2 was developed as follows:

**Hypothesis** **H1-2.** *Person-group fit will have a positive impact on university PE Teacher performance.*

#### 2.1.3. Person-Organization Fit

Chatman [32] defined person-organization fit (P-O fit) as the congruency between patterns of organizational values and patterns of individual values, and the P-O fit affects employees’ work attitudes and behaviors in a positive way. It is noted that P-O fit originated from Schneider’s [33] ASA (attraction-selection-attrition) model, in which employees are attracted to an organization whose members are similar to themselves in terms of values, goal, culture, and other attributes. Through the recruitment process and personal selection, new employees who fit in the organization and share similar core values and goals with existing employees are more likely to stay. Those who do not fit are more likely to leave [34].

Gregory, Albritton, and Osmonbekov [35] conducted an empirical research with 116 university faculty and staff from the Western United States, and concluded that their perceived P-O fit is a significant predictor of their job satisfaction and in-role performance. Similarly, Afsar, Badir, and Khan [25] reported that employees’ perceived P-O fit positively affected their innovative work behaviors. Accordingly, Hypothesis 1−3 was proposed as follows:

**Hypothesis** **H1-3.** *Person-organization fit will have a positive impact on university PE Teacher performance.*

#### 2.1.4. Person- Supervisor Fit

Person-supervisor fit (P-S fit), refers to the similarity between individual and supervisor personality dimensions, values, and goals [28]. P-S fit is nothing like leader-member exchange (LMX). Although both theories are about the exchange between employees and supervisors, P-S fit puts emphasis on an emotional tie between subordinate and supervisor, while LMX emphasizes the work relationship the leader and their members develop.

According to self-categorization theory, individuals have a tendency to interact with people with similar traits and make a sense of group during socialization [36]. When subordinates and supervisors have similar personal characteristics and goals, it is easy to make a successful team and create a good atmosphere in the office. Employees working in a pleasing environment are less likely to commit misconduct to destroy their organization [37]. In fact, they are more likely to work overtime and take extra responsibilities by exhibiting organizational citizenship behaviors [38]. Accordingly, Hypothesis 1−4 was developed as follows:

**Hypothesis** **H1-4.** *Person- Supervisor Fit will have a positive impact on university PE Teacher performance.*

### 2.2. Organizational Support and Performance

Eisenberger, Huntington, Hutchison, and Sowa [16] stated that social exchange is the only way to build mutual responsibility, interests and trust. When employees perceive the organizational support such as care, appreciation, and recognition, they are motivated and inspired to be highly dedicated and show stronger support and commitment to their organization. Yuan, Chen, and Xiao [39] indicated that perceived organizational support (POS) has some positive influences: (a) POS satisfies employees’ social emotional needs and make them feel respected, cared for and recognized. POS encourages group members to work together and to appreciate their individual roles in the society; (b) POS emphasizes the belief, which is a must to high organization performance, that hard work is the key to success.

Sun [40] reported that POS plays an important role in employee job performance. The study’s findings suggest that employees with high POS at some production department in an organization are more dedicated to their job, and therefore have more satisfying job performance [41]. Similarly, sales employees with high POS receive positive feedback which in turn increases their job performance [42]. Based on 70 research findings, POS is positively related with job performance. As a result, Hypothesis 2 was formulated as follows:

**Hypothesis** **H2.** *Organizational Support will have a positive impact on university PE Teacher performance.*

### 2.3. Moderating Effects of Organizational Support on Person-Environment Fit and Performance

In addition to the abovementioned study findings, organizational support as a variable is found to have direct positive influence on employees’ positive behaviors. Organizational support was also considered as a moderator variable in several studies [43,44]. Organizational support is a resource which can not only reduce the effect of stress an employee can feel [45] but also minimize the impact that workplace violence has on job satisfaction and organizational commitment [46]. In other words, organizational support can be a moderator between workplace bullies, job performance, and turnover intention [47].

According to Erdogan and Enders [48], organizational support has reinforcement effect that reinforces the job satisfaction and job performance employees perceive from LMX. Witt and Carlson [49] also stated that people with much work experience facing high levels of work-family conflict are likely to maintain their work motivation to exert force and sustain effective performance if they receive considerable organizational support, which refers to more resources, mutual responsibilities, and a perspective that constant effort equals desired results.

However, no research has been done to explore the mediating effect of organizational support on the relationship between person-environment fit and job performance. Based on prior inferences, the present study considers organizational support as an upper-level variable, and investigates the mediating effect of organizational support on the relationship between person-environment fit and job performance. Accordingly, four hypotheses were developed as follows.

**Hypothesis** **H3-1.** *Organizational Support will have a positive moderating effect on person-job fit and university PE Teacher performance.*

**Hypothesis** **H3-2.** *Organizational Support will have a positive moderating effect on person-group fit and university PE Teacher performance.*

**Hypothesis** **H3-3.** *Organizational Support will have a positive moderating effect on person-organization fit and university PE Teacher performance.*

**Hypothesis** **H3-4.** *Organizational Support will have a positive moderating effect on person-supervisor fit and university PE Teacher performance.*

**Figure 1:**
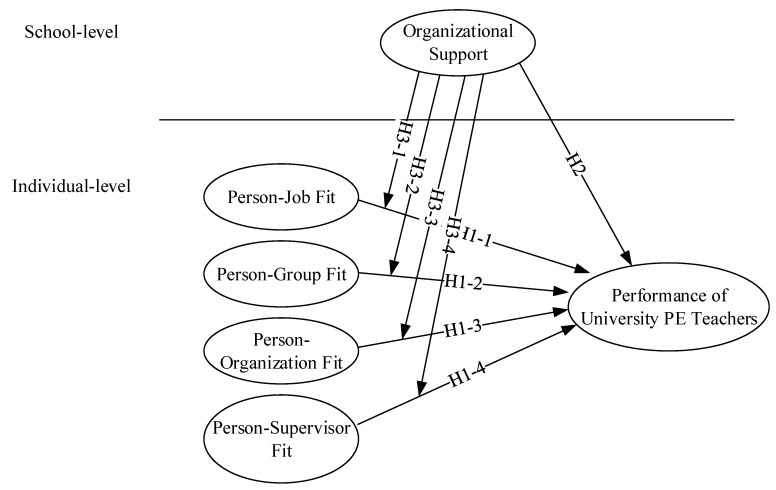
Research hypotheses.

Model depiction of the study hypotheses based on previous inferences are presented on Figure 1.

## 3. Method

### 3.1. Participants

The research participants were physical education teachers from 55 comprehensive universities and technical universities with at least 10 or more PE teachers. In order to have high response rate, the researcher asked one PE teacher from each university to conduct the survey. The assigned PE teacher explained the study purpose to the participants and obtained their consent before actually distributing the survey questionnaires to the participants. The assigned PE teacher left while the participants were filling out the survey. The study was reviewed and approved by National Cheng Kung University’s Institutional Review Board. Cluster sampling was used for data collection. A total of 680 survey questionnaires were sent to PE teachers from 55 universities. A total of 447 questionnaires from 55 universities were collected for data analysis, with a valid response rate of 65.74%.

Table 1 presents the descriptive statistics of participants. Research participants were comprised of 301 male teachers (67.3%) and 146 female teachers (32.7%). Regarding age, 22 participants (4.9%) were under 35, 256 (57.3%) belonged to the 36−50 year-old group, and 169 (37.8%) were above 51. Concerning educational background, demographic information indicated that there were 2 (0.4%) participants with a college diploma, 52 (11.6%) with a university degree, 264 (59.1%) with a master’s degree, and 126 (28.9%) with a doctoral degree.

Some research participants took not only teaching roles but also administrative responsibilities. It was noted that 166 (37.1%) participants had to take care of administrative work while 281 (62.9%) did not. In terms of work title, 83 participants (18.6%) were instructors, 139 (31.1%) were assistant professors, 160 (35.8%) were associate professors, and 65 (14.5%) were professors or distinguished professors. Out of 447 PE teachers, 172 (38.5%) were teaching in public universities while 275 (61.5%) were teaching in private ones. There were 25 participants (5.6%) with 5-year teaching experience or less, 51 (11.4%) with 6−10 years of experience, 142 (31.8%) with 11−20 years of experience, and 229 (51.2%) with 21-year teaching experience or more. Sixty-eight participants (15.2%) were not married, and 379 (84.8%) were married.

### 3.2. Research Instruments

The four scales used for data analysis are presented along with demographic information below.

### 3.3. Person-Environment Fit Scale

Person-environment fit scale was a modified version of Chuang and Lin [34] and Huang [50], having a total of 16 items which are divided into four groups consisting of four items each: the Person-Job Fit Scale (PJFS), the Person-Organization Fit Scale (POFS), the Person-Group Fit Scale (PGFS), and the Person-Supervisor Fit Scale (PSFS). All scales were measured using a 7-point Likert scale, ranging from 1 = strongly disagree to 7 = strongly agree.

Four questions were asked in each individual group. One of the PJFS questions reads, “Your personality traits match those required by the job.” The PGFS sample question reads, “Your emphasis on your work value match your group members’ (not including your supervisor’s).” The POFS asks “Your organization’s culture matches your personal work value”.

The total variance explained by the four factors in the factor analysis was found to be 78.58%, with factor loading for each item falling between 0.58 and 0.87. The internal consistency showed that Cronbach alpha of 0.83 for PJFS, 0.88 for PGFS 0.93 for PSFS, and 0.95 for POFS. In addition, achieved Cronbach alpha of 0.94 confirmed high internal consistent reliability for this person-environment fit survey questionnaire.

The results from confirmatory analysis show that all measurements fit the requirement of reliability and validity (χ2 = 341.85, df = 98, χ2/df = 3.49, CFI = 0.98, GFI = 0.91, NFI = 0.98, IFI = 0.98, RMSEA = 0.07).

### 3.4. University PE Teacher Performance Scale

The study used the university PE teacher performance scale created by Chang, Lin, Chia, and Yang [7]. The scale comprised seven items divided into three types of job responsibility: teaching performance, research performance, administration and student counselling performance. All scales were measured using a 7-point Likert scale, ranging from 1 = strongly disagree to 7 = strongly agree. The factor analysis showed that the total variance explained of one factor was 69.22%, with factor loadings of seven items falling between 0.65 and 0.85. Achieved Cronbach’s alpha of 0.89 confirmed high internal consistent reliability for the scale. The results from confirmatory analysis show that all measurements fit the requirement of reliability and validity (*χ*^2^ = 48.74, df =11, *χ*^2^/df = 4.43, CFI = 0.99, GFI = 0.97, NFI = 0.98, IFI = 0.99, RMSEA = 0.09).

### 3.5. Organizational Support Scale

Based on the suggestion of Eisenberger, Stinglhamer, Vandenberghe, Sucharski, and Rhoades [51], and Shanock and Eisenberger [52], the study utilized Eisenberger et al.’s [16] six topics of the survey of perceived organizational support (SPOS). Following the approaches of Rhoades and Eisenberger [18], the study replaced “organization” with “my direct supervisor” to better fit the study purpose. The scale was measured using a 7-point Likert Scale (1 = strongly disagree, 7 = strongly agree). The factor analysis results indicate that the total variance explained of one factor was 75.83%. Factor loading of all items fell within 0.70 and 0.89. In addition, the achieved Cronbach’s alpha of 0.92 confirmed high internal consistent reliability for the scale. The results from confirmatory analysis show that all measurements fit the requirement of reliability and validity (*χ*^2^ = 27.19, df = 7, *χ*^2^/df = 3.88, CFI = 0.99, GFI = 0.98, NFI = 0.99, IFI = 0.99, RMSEA = 0.08).

### 3.6. Demographic Information or Control Variables

Previous studies on PE teacher performance found that position and teaching experience have significant influence on self-efficacy and job involvement [53]. Marriage, age, and administrative work had some impacts on teaching performance [54]. As age and teaching experience are highly related, this study selected teaching experience as a control variable. School type (public or private), gender, position, education, teaching experience, administrative work, and marriage were the seven control variables in this particular study.

### 3.7. Data Analysis

Statistical analysis was performed using SPSS 20.0 for Windows and HLM 7.0. Given the significance level of 0.05, the hypothesis testing was conducted using descriptive statistics, Pearson correlation, and hierarchical liner modeling (HLM).

## 4. Results

### 4.1. Correlations and Descriptive Statistics

Klein, Dansereau, and Hall [55] proposed that the appropriateness of aggregating individual outcomes to the group level has to be determined prior to conducting HLM analyses. In this study, organizational support was a shared construct, and data were collected from each individual teacher. According to James, Demaree, and Wolf [56], the r_wg_ index is a measure of interrater agreement, and is typically used to determine the appropriateness of aggregating data to higher levels of analysis. A 0.70 criterion is commonly used. R_wg_ values equal to or greater than 0.70 demonstrate high consistency within groups and justify the aggregation within the group. In addition, Bliese [57] suggested that ICC (1) represents the percentage of variance between groups, and ICC (2) represents the reliability of the group mean scores. According to Castro [58], the ICC (1) value has to be greater than 0.07, while the ICC (2) value has to greater than 0.50. Both ICC (1) = 0.37 and ICC (2) = 0.82 for organization support meet the statistical requirement. The average r_wg_ for organizational support was 0.76 (range 0.70 to 0.79, SD = 0.02). Each value was greater than 0.70, indicating that data combined from organizational support at an individual level can be aggregated to school levels of analysis.

Descriptive statistics of individual-level variable and school-level variable and variable analysis (means score and standard deviation) are presented in Table 2.

### 4.2. Hypothesis Testing

Nine hypotheses are developed throughout the study and tested using HLM. The results are as follows:

#### 4.2.1. Null Model Analysis

A cross-level analysis was conducted to explore the impact of variables at individual level and at school level on university PE teacher performance. Chi-square test in the null model presented in Table 3 indicated that between-group variance in PE teacher performance is significant (χ^2^ = 173.93, df = 54, *p* < 0.001), and ICC (1) is 0.215, indicating that school difference accounts for 21.5% of the total variance of PE teacher performance. As a result, HLM was employed in null model analysis and the level-1 and level-2 models are expressed in the following:

**Level-1 Model**

PE teacher performance *_ij_*=*β*_0*j*_ + *r_ij_*

**Level-2 Model**

*β*_0*j*_=*γ*_00_ + *u*_0*j*_

#### 4.2.2. Hierarchical Linear Modeling (HLM) Analysis of PE Teacher Performance

The level-1 and level-2 models of the HLM analysis are shown in the following:

**Level-1 Model**

PE teacher performance*_ij_* = *β*_0*j*_ + *β*_1j_*(school type*_ij_*) + *β*_2j_*(Gender*_ij_*) + *β*_3j_*(*RANKING_ij_*) + *β*_4j_*(Education*_ij_*) + *β*_5j_*(Administrative work*_ij_*) + *β*_6j_*(Teaching experience*_ij_*) + *β*_7j_*(Marriage*_ij_*) + *β*_8j_*(Person-Job Fit *_ij_*) + *β*_9j_*(Person-Group Fit *_ij_*) + *β*_10j_*(Person-Supervisor Fit *_ij_*) + *β*_11j_*(Person-Organization Fit *_ij_*) + *r_ij_*

**Level-2 Model**

*β*_0*j*_ = *γ*_00_ + *γ*_01_*(organizational support *_j_*) + *u*_0*j*_

*β*_1*j*_ = *γ*_10_

*β*_2*j*_ = *γ*_20_

*β*_3*j*_ = *γ*_30_

*β*_4*j*_ = *γ*_40_

*β*_5*j*_ = *γ*_50_

*β*_6*j*_ = *γ*_60_

*β*_7*j*_ = *γ*_70_

*β*_8*j*_ = *γ*_80_ + *γ*_81_*( organizational support *_j_*)

*β*_9*j*_ = *γ*_90_ + *γ*_91_*( organizational support *_j_*)

*β*_10*j*_ = *γ*_100_ + *γ*_101_*( organizational support*_j_*)

*β*_11*j*_ = *γ*_110_ + *γ*_111_*( organizational support*_j_*)

Notice that in order to perform HLM analysis, school type, person-job fit, person-group fit, person-organization fit, and person-supervisor fit have been centered around the group mean and organizational support has been centered around the grand mean.

**Figure 2:**
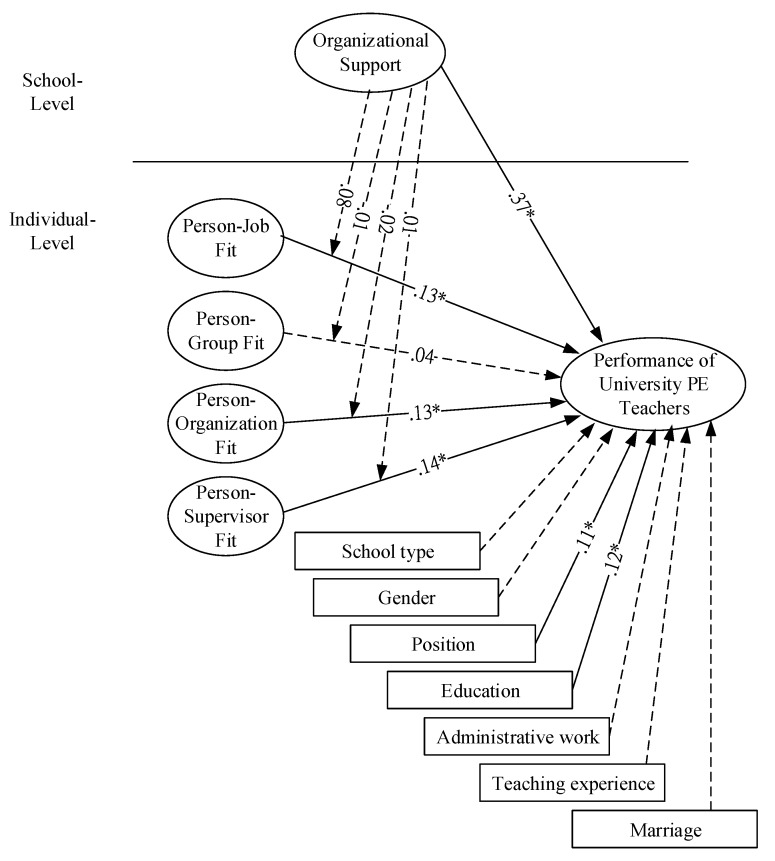
Results of hierarchical linear modeling (HLM). * *p* < 0.05

Table 4 and Figure 2 present the HLM analysis results. It shows that the impacts of education and administrative work on PE teacher performance were significant. Teacher position (*γ*_20_ *=* 0.11, *t* = 3.32, *p* < 0.05) had positive significant impacts on performance, indicating that higher position results in better performance. Education (*γ*_30_ = 0.12, *t* = 2.67, *p* < 0.05) also had positive significant impacts on performance, meaning that PE teachers who have higher levels of education have higher performance.

Concerning the impacts of P-J fit, P-G fit, P-S fit and P-O fit of university PE teachers on their performance at an individual level, the research showed that P-J fit (*γ*_80_ *=* 0.13, *t* = 2.57, *p* < 0.05) positively affects teacher performance, indicating that the higher the P-J fit level, the better the teacher performance. Similar results were found with P-S fit (*γ*_100_ *=* 0.13, *t* = 3.29, *p* < 0.05) and P-O fit (*γ*_110_ *= 0*.14, *t* = 3.22, *p* < 0.05), suggesting that higher fit perceptions lead to better teacher performance. All results are shown in Table 4.

The cross-level analysis showed that organization support at school level has positive impacts on PE teacher performance at individual level (*γ*_01_ = 0.37, *t* = 4.01, *p* < 0.05). Teachers who received strong organizational support across various universities were more likely to have better performance. However, the moderating effect of organizational support at the school level on the relationship between P-E fit and university PE teacher performance was not found to exist. The results are presented in Table 4.

## 5. Discussion

### 5.1. Person-Environment Fit and Performance

Our results show that the person-job fit, person-organization fit and person-supervisor fit had significant impacts on performance but not person-group fit, which supports the findings of the meta-study of the relationships of person–environment fit dimensions with work attitudes and performance across East Asia, Europe, and North America [59], complying with East Asian culture, that interdependency and submission to organizations other than personal contributions have significant impacts on performance.

### 5.2. Organizational Support and Performance

Perceived organizational support is considered to be a resource capable of positively influencing performance by reducing stressors and encouraging commitment. According to the cross-level analysis, our study showed that organizational support had significant impacts on PE teachers’ performance (*γ*_01_ = 0.37, *t* = 4.01) which also support the findings of Byrne and Hochwarter [60] indicating the organizations’ support on employees can affect their performance in a positive way.

### 5.3. Moderating Effect of Organizational Support between Person-Environment Fit and Performance

According to our HLM analysis, there is no significant evidence showing the moderating effect (or enhancing effect) on the relationship between person-environment fit and performance. This may come without surprise since PE in oriental society is not treated with due respect. In oriental society, parents most care about children’s academic achievements, leading to deprivation of PE courses, and sometimes the courses are replaced by some academic-related subjects, leaving the PE teachers in an embarrassing situation. In particular, many of them do have well-trained skills but are not very active in engaging in academic production, and hence lack organizational support. They deserve organizations’ attention and support.

## 6. Contribution and Implication for Application

### 6.1. Academic Contribution

There has been very little research on university PE teacher performance, let alone studies that analyze what might affect teacher performance. This current study aims to investigate the impact of P-E fit on university PE teacher performance, and four dimensions of P-E fit were all included. Data analysis with 447 PE teachers from 55 universities in Taiwan indicated that P-J fit, P-S fit, and P-O fit all had a significant positive impact on PE teacher performance. The research results also suggest that the fit of skill, value, and characteristics between an individual and organization can be an important factor affecting university PE teacher performance.

Furthermore, when recruiting new PE teachers, universities should pay extra attention to whether the candidate’s professional skills and knowledge are a good match for the teaching job. Candidate’s perceptions about value and culture are to be considered, too. If candidates and organizations share the same values, candidates are more likely to stay, or quit otherwise [34]. Similar values, goals and personal characteristics are some key factors to make a team in which individuals interact smoothly to the benefit of school organization [37]. Hoecklin further stated that in oriental culture, supervisors are typically more influential to their subordinates as compared to those from a western cultural background. Thus, P-S fit has some special meaning in Chinese oriental culture [26].

The present study also investigated school-level organizational support and conducted a cross-level analysis. The findings show that organizational support positively and significantly affect university PE teacher performance; nevertheless, the moderating effect of organizational support on the relationship between P-E fit and PE teacher performance was not found.

Unlike early studies that conducted analysis only at the individual level [54], in order not to miss out any possible variables this particular study included school-level variables. The findings are consistent with the previous studies that showed that teachers perceiving more organizational support tend to have more positive work attitudes and better job performance [18,41]. With increased perception of organizational support, teachers consider themselves as organization’s “insiders”, which in turn motivates them to be more willing to offer positive feedback to the school [17]. In order to retain teachers and encourage them to give back, universities should understand and satisfy teachers’ needs [61].

### 6.2. Managerial Implications

The findings indicate that consistency of PE teachers’ personal skills to job requirements has to be ensured in order to enhance university PE teacher performance. This consistency is especially important in Taiwan, as under Taiwan’s education system it is not easy to get rid of incompetent teachers. Prior to official recruitment, several things to consider include: the candidate’s role and responsibilities in the university and respective department, personal skills, work behaviors and attitudes, and personal values, goals and cultures. While it is important to assess a candidate’s personal qualities, it is even more important to put emphasis on matching individuals to organization’s culture, value, and goals. A good fit between organizational characteristics and individual focus has an impact on teachers’ involvement and retention. Since PE teachers in Taiwan universities typically have a close work relationship with their supervisors, it is important for supervisors to become charismatic leaders and create a work culture to improve PE teachers’ job performance.

## 7. Research Limitations and Recommendations for Future Studies

In Taiwan universities, the work content of PE teachers typically includes teaching, research, and counseling service and administrative work. It should be noted that teachers from western countries may have different areas of responsibilities, and assessment tools are constructed using different indicators; and thus, this research conclusion should be applied with caution. It is also argued that factors that affect teachers’ performance can have a different degree of impact. For instance, P-S fit perhaps has greater impacts on administrative work and student counseling services, but less impact on research performance. Therefore, it is recommended that future study can be conducted to separately analyze teaching performance, research performance, and administration and student counseling services. This approach may especially work better with universities that categorize teachers based on their areas of expertise, and should help future research better identify factors that have impacts on teaching performance and research performance. Recent research on the impacts of an innovative teaching environment on innovative teaching performance is a good example of this approach.

## 8. Conclusions

Our results show that the three dimension of P-E fit (P-J fit, P-O fit and P_S fit) had positive significant impacts on university PE teachers’ performance. The cross-level analysis found organizational support had direct positive impacts on performance but there was no significant moderating (enhancing) effect on the relationship of the P-E fit to performance. Among seven individual-level control variables (school type, gender, teaching experience, position, administrative work, education, and marriage), position and teaching experience were reported to have a significant positive impact on teacher performance.

## Figures and Tables

**Table 1:** Descriptive statistics of participants.

Variables	Frequency	%	Variables	Frequency	%
Gender			Work title		
Female	146	32.7	Instructor	83	18.6
Male	301	67.3	Assistant professor	139	31.1
Age			Associate professor	160	35.8
35-	22	4.9	Professors or distinguished professors	65	14.5
36−50	256	57.3	University type		
51+	169	37.8	Public university	172	38.5
Education			Private university	275	61.5
College diploma	2	0.4	Teaching experience		
University degree	52	11.6	5-years and less	25	5.6
Master’s degree	264	59.1	6-10 years	51	11.4
Doctoral degree	129	28.9	11-20 years	142	31.8
Administrative work			More 21 years	229	51.2
Yes	166	37.1	Married		
No	281	62.9	No	68	15.2
			Yes	379	84.8

**Table 2:** Summary statistics and Pearson’s coefficient of correlation of individual- and school-level variables.

Individual-Level Variables	M	SD	1	2	3	4	5	6	7	8	9	10	11
1. School type (public/private)	1.62	0.487	1.00										
2. Gender	1.33	0.470	0.06	1.00									
3. Teaching experience	20.33	8.794	0.20 ^*^	−0.01	1.00								
4. Position	2.46	0.955	−0.09	−0.04	0.42 ^*^	1.00							
5. Administrative work	1.63	0.484	0.11 ^*^	0.01	0.18 ^*^	0.00	1.00						
6. Education	3.16	0.630	−0.27 ^*^	−0.02	−0.35 ^*^	0.18 ^*^	−0.12 ^*^	1.00					
7. Marriage	1.85	0.360	0.07	−0.18 ^*^	0.23 ^*^	0.19 ^*^	0.05	−0.04	1.00				
8. P-J Fit	3.74	0.74	−0.11 ^*^	−0.07	0.11 ^*^	0.15 ^*^	−0.04	0.04	0.00	1.00			
9. P-G Fit	3.38	0.74	−0.08	0.07	0.01	0.01	0.00	0.03	0.03	0.48 ^*^	1.00		
10. P-S Fit	3.30	0.93	−0.11 ^*^	−0.04	−0.02	0.02	−0.04	0.03	0.01	0.56 ^*^	0.52 ^*^	1.00	
11. P-O Fit	3.43	0.87	−0.09	0.02	0.03	0.10 ^*^	−0.04	0.03	0.00	0.59 ^*^	0.60 ^*^	0.56 ^*^	1.00
12. Performance	3.71	0.63	−0.05	0.02	0.14 ^*^	0.26 ^*^	−0.04	0.13 ^*^	0.03	0.43 ^*^	0.35 ^*^	0.42 ^*^	0.46 ^*^
School-level variables													
Organizational Support	3.87	0.46											

Note: n = 447 at individual-level variables; n = 55 at school-level variables; SD = standard deviation; * *p* < 0.05.

**Table 3:** Null model analysis.

Random Effect	Standard Deviation	Variance Component	*d.f.*	χ^2^	*p*-value
INTRCPT1, *u*_0_	0.293	0.086	54	173.93	0.001
level-1, *r*	0.561	0.314			

**Table 4:** Hierarchical linear modeling (HLM) analysis of PE teacher performance.

Fixed Effect	Coefficient	Standard Error	*t*-Ratio	Approx. *d.f.*	*p*-Value
For INTRCPT1, *β*_0_					
INTRCPT2, *γ*_00_	2.99	0.24	12.50 *	53	0.001
organizational support, *γ*_01_	0.37	0.09	4.01 *	53	0.001
For School type slope, *β*_1_					
INTRCPT2, *γ*_10_	−0.59	0.50	−1.17	377	0.244
For gender slope, *β*_2_					
INTRCPT2, *γ*_20_	0.02	0.05	0.41	377	0.682
For Position slope, *β*_3_					
INTRCPT2, *γ*_30_	0.11	0.03	3.32	377	0.001
For Education slope, *β*_4_					
INTRCPT2, *γ*_40_	0.12	0.05	2.67	377	0.008
For Administrative work slope, *β*_5_					
INTRCPT2, *γ*_50_	−0.07	0.05	−1.37	377	0.173
For Teaching experience slope, *β*_6_					
INTRCPT2, *γ*_60_	0.01	0.01	1.80	377	0.073
For Marriage slope, *β*_7_					
INTRCPT2, *γ*_70_	0.01	0.07	0.13	377	0.895
For P-J Fit slope, *β*_8_					
INTRCPT2, *γ*_80_	0.13	0.05	2.57	377	0.010
SPOS, *γ*_81_	0.08	0.12	0.67	377	0.502
For P-G Fit slope, *β*_9_					
INTRCPT2, *γ*_90_	0.04	0.05	0.84	377	0.403
SPOS, *γ*_91_	−0.01	0.10	−0.14	377	0.886
For P-S Fit slope, *β*_10_					
INTRCPT2, *γ*_100_	0.13	0.04	3.29	377	0.001
SPOS, *γ*_101_	−0.02	0.09	−0.18	377	0.858
For P-O Fit slope, *β*_11_					
INTRCPT2, *γ*_110_	0.14	0.04	3.22	377	0.001
SPOS, *γ*_111_	0.01	0.10	0.13	377	0.894
